# MyD88 Is Required for Protection from Lethal Infection with a Mouse-Adapted SARS-CoV

**DOI:** 10.1371/journal.ppat.1000240

**Published:** 2008-12-12

**Authors:** Timothy Sheahan, Thomas E. Morrison, William Funkhouser, Satoshi Uematsu, Shizou Akira, Ralph S. Baric, Mark T. Heise

**Affiliations:** 1 Department of Microbiology and Immunology, University of North Carolina at Chapel Hill, Chapel Hill, North Carolina, United States of America; 2 Department of Epidemiology, University of North Carolina at Chapel Hill, Chapel Hill, North Carolina, United States of America; 3 Department of Genetics, University of North Carolina at Chapel Hill, Chapel Hill, North Carolina, United States of America; 4 Carolina Vaccine Institute, University of North Carolina at Chapel Hill, Chapel Hill, North Carolina, United States of America; 5 Department of Pathology, University of North Carolina School of Medicine, Chapel Hill, North Carolina, United States of America; 6 WPI Immunology Frontier Research Center, Laboratory of Host Defense, Osaka University, Suita, Osaka, Japan; National Institutes of Health, United States of America

## Abstract

A novel human coronavirus, SARS-CoV, emerged suddenly in 2003, causing approximately 8000 human cases and more than 700 deaths worldwide. Since most animal models fail to faithfully recapitulate the clinical course of SARS-CoV in humans, the virus and host factors that mediate disease pathogenesis remain unclear. Recently, our laboratory and others developed a recombinant mouse-adapted SARS-CoV (rMA15) that was lethal in BALB/c mice. In contrast, intranasal infection of young 10-week-old C57BL/6 mice with rMA15 results in a nonlethal infection characterized by high titer replication within the lungs, lung inflammation, destruction of lung tissue, and loss of body weight, thus providing a useful model to identify host mediators of protection. Here, we report that mice deficient in MyD88 (MyD88^−/−^), an adapter protein that mediates Toll-like receptor (TLR), IL-1R, and IL-18R signaling, are far more susceptible to rMA15 infection. The genetic absence of MyD88 resulted in enhanced pulmonary pathology and greater than 90% mortality by day 6 post-infection. MyD88^−/−^ mice had significantly higher viral loads in lung tissue throughout the course of infection. Despite increased viral loads, the expression of multiple proinflammatory cytokines and chemokines within lung tissue and recruitment of inflammatory monocytes/macrophages to the lung was severely impaired in MyD88^−/−^ mice compared to wild-type mice. Furthermore, mice deficient in chemokine receptors that contribute to monocyte recruitment to the lung were more susceptible to rMA15-induced disease and exhibited severe lung pathology similar to that seen in MyD88^−/−^mice. These data suggest that MyD88-mediated innate immune signaling and inflammatory cell recruitment to the lung are required for protection from lethal rMA15 infection.

## Author Summary

In 2002, a new human coronavirus (CoV), termed SARS-CoV, emerged in southern China from coronaviruses circulating within live animals sold for food. Due to the ease and speed of human global travel, this new respiratory virus rapidly spread worldwide, illustrating the need to better understand how these viruses cause disease and how the immune system responds to infection. SARS-CoV infection of the human lower respiratory tract caused an atypical pneumonia characterized by viral replication in lung tissue and lung inflammation visible by chest X-ray. To identify how the immune system responds to and provides protection from SARS-CoV infection, we have developed a mouse model that mimics many aspects of SARS-CoV disease in humans. Utilizing this mouse model, we discovered that a host gene, termed MyD88, is required to control SARS-CoV replication and spread in lung tissue and for protection from death. In addition, MyD88-dependent functions were required for early immune and inflammatory responses in the lung following SARS-CoV infection, and the absence of these early responses correlated with severe SARS-CoV-induced disease and death. Our studies identify host immune responses that provide protection from SARS-CoV infection and provide valuable insight toward the development of successful antiviral therapies.

## Introduction

In 2003, a novel coronavirus, SARS-CoV, emerged from zoonotic pools of virus in China to cause a global outbreak of Severe and Acute Respiratory Syndrome (SARS) affecting 29 countries, causing over 8000 human cases and greater than 700 deaths [1]–[3]. The clinical course of SARS-CoV disease in humans is characterized by fever, non-productive cough, and malaise culminating in lung infiltrates visible by X-ray and an atypical pneumonia [4]–[8]. Immunologically, SARS-CoV infection of humans generates a cytokine/chemokine storm where elevated levels of IP-10, MIP1-α, and MCP-1 are detected within the blood [9]. Histological examination of lung tissue in terminal SARS-CoV cases revealed SARS antigen primarily within bronchiolar epithelium, Type I and II alveolar pneumocytes, and less frequently within macrophages and lymphocytes in the lung, suggesting a roll for multiple cell types in SARS-CoV pathogenesis [10],[11].

Though clinical and epidemiological data from the epidemic and reemergence has provided insight into the molecular pathogenesis of SARS-CoV infection, thorough studies of virus and host interactions have been hampered by the lack of animal models that fully recapitulate human disease. C57BL/6 mice infected with the epidemic strain, SARS Urbani, do not show any overt signs of disease but there is virus replication in the lung (10^7^TCID_50_/g 3dpi), induction of a number of proinflammatory chemokines, and viral clearance even in the absence of T, B, and NK cells, suggesting that innate immunity alone is required for the clearance of SARS Urbani within this acute model of SARS-CoV replication [12]. The newly developed mouse adapted SARS-CoV, MA15, differs from Urbani in 6 amino acids and infection of young or senescent BALB/c mice with either MA15 or recombinant MA15 (rMA15) results in high virus titers in the lung, pulmonary pathology, and 100% mortality resembling the pathogenesis of the most severe human cases of SARS-CoV [5],[10],[13]. Unfortunately, a SARS-CoV mouse model does not yet exist that recapitulates the less severe pathogenesis and recovery seen in a majority of the human cases. Moreover, a model of SARS-CoV pathogenesis with both disease and convalescence would allow for the elucidation of pathways involved in the innate or adaptive protective response to infection.

Toll-like receptors (TLRs) are cellular receptors that recognize molecular signatures of pathogens and initiate an inflammatory signaling cascade that is critical to the innate immune response [14]. Myeloid differentiation primary response gene 88 (MyD88) is a key adaptor protein for most TLR-dependent inflammatory signaling pathways as well as IL-1R1, IL-18R1 and IFNγR1 signaling pathways [14] MyD88 interacts with a variety of cellular proteins leading to the activation of NF-κB, JNK, and p38 and the induction of inflammatory cytokines, chemokines, and type I interferons [14]. The role of MyD88 in the host response to viral infection has been investigated for a number of viral pathogens. These studies have indicated that MyD88 is crucial for the response to some viral infections, while it appears dispensable for others. For example, MyD88 signaling is not required for clearance of reovirus infection after peroral inoculation of mice [15]. In contrast, MyD88^−/−^ mice infected with respiratory syncytial virus (RSV), vesicular stomatitis virus (VSV), or lymphocytic choriomeningitis virus (LCMV) results in more severe disease [16]–[19]. Though TLR7/MyD88/IFNα dependent signaling has been implicated as important in the pathogenesis of a related coronavirus, mouse hepatitis virus (MHV), the role of MyD88 signaling in SARS-CoV pathogenesis has not yet been investigated [20].

In this study, we describe a novel C57BL/6 mouse model of rMA15 acute pathogenesis characterized by high titer virus replication within the lung, induction of inflammatory cytokines and chemokines, and immune cell infiltration within the lung. WT mice display signs of disease that include 12–15% loss of body weight by 3 dpi and lung pathology, however, these mice recover from infection by 6 dpi. Furthermore, we demonstrate a protective role for MyD88-dependent regulation of innate and inflammatory immune responses in this model of rMA15 pathogenesis. MyD88^−/−^ mice infected with rMA15 have significantly higher and prolonged virus titers in the lung, exhibit a severe delay in host immune and inflammatory responses, including monocyte/macrophage recruitment to the lung, and ultimately succumb to infection. In addition, mice deficient in chemokine receptors that regulate the recruitment of inflammatory leukocytes to the lung were also more susceptible to rMA15 infection. These data suggest that a failure or delay in MyD88 inflammatory signaling and a concurrent delay in inflammatory monocyte/macrophage recruitment to the lung during acute infection results in exacerbated SARS-CoV disease. This novel mouse model of acute SARS-CoV pathogenesis could be extended to the investigation of many other components of the innate immune response in order to form a more comprehensive view of SARS-CoV pathogenesis, which may guide the rational design of antiviral therapies.

## Results

### MyD88^−/−^ mice are highly susceptible to rMA15 infection

**Figure 1:**
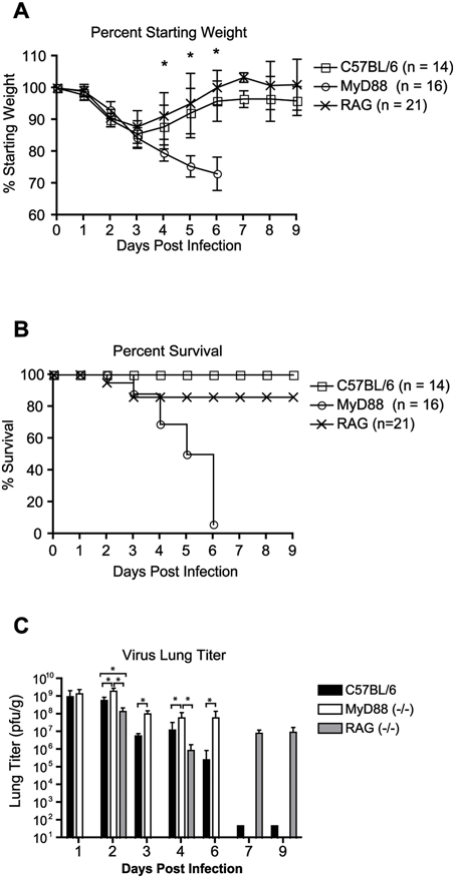
MyD88^−/−^ mice are highly susceptible to rMA15 infection and experience higher levels of virus replication within the lung as compared to WT mice. 10 week-old C57BL/6 WT (n = 14), RAG-1^−/−^ (n = 21), or MyD88^−/−^ (n = 16) mice were infected intranasally with 10^5^ pfu rMA15. (A) Mice were monitored for changes in body weight at 24 h intervals. Each data point represents the mean ± the standard deviation. Asterisks indicate statistical significance by the non-parametric Mann-Whitney test with a P<0.05. (B) Mice were monitored for mortality. Data is expressed as a percent survival value. (C) WT, MyD88^−/−^ or RAG-1^−/−^ mice were infected intranasally with 10^5^ pfu rMA15. Lung tissues from 3–6 mice per strain were harvested on 1, 3, 4, 6, and 9 dpi. Lung tissues were homogenized in DPBS and virus titers within clarified supernatants were assessed by plaque assay. Mean titers are displayed and error bars represent 1 standard deviation. *, P<0.05 as determined by the Mann-Whitney test.

Since previous studies suggested MyD88-dependent inflammatory signaling was important for protection from severe disease caused by VSV, LCMV, and RSV, we evaluated the importance of MyD88 signaling in SARS-CoV pathogenesis [16],[18],[19],[21]. To assess the contribution of innate and adaptive immune responses in SARS-CoV-induced disease, age matched C57BL/6 (WT) (n = 14), congenic RAG-1^−/−^ (n = 21), and congenic MyD88^−/−^ (n = 16) mice were infected intranasally with 10^5^ pfu of recombinant mouse-adapted SARS-CoV (rMA15) and monitored for virus-induced morbidity and mortality. Infection of WT, RAG-1^−/−^, or MyD88^−/−^ mice resulted in weight loss beginning at day 2 post-infection (Fig. 1A). Infected WT and RAG-1^−/−^ mice lost 14±4% and 9±4% of starting body weight by 3 dpi, respectively, and returned to starting body weights by 5–6 dpi, indicating that the mice had recovered from rMA15 induced disease. In contrast to WT and RAG-1^−/−^ mice, which began to recover weight after 3 dpi, infected MyD88^−/−^ mice continued to lose weight after 3 dpi creating a significant weight disparity between WT and MyD88^−/−^ mice (WT vs. MyD88 percent weight p = <0.05 at 4, 5, and 6 dpi). While 100% of WT and 86% of RAG-1^−/−^ mice survived the infection, >90% of MyD88^−/−^ mice (n = 16) succumbed to infection by 6 dpi (Fig. 1B). MyD88 plays a critical role in proinflammatory signaling following stimulation of all known TLR's, except TLR3, as well as IL1R and IL18R. Interestingly, 100% of IL-1R1- or IL-18R1-deficient mice survived infection by rMA15 (data not shown) indicating that the genetic absence of either receptor did not recapitulate the lethal phenotype observed following infection of MyD88^−/−^ mice. These findings indicate that i) adaptive immunity does not play a major role in protection from lethal SARS-CoV infection in mice, ii) the genetic absence of MyD88 significantly enhances SARS-CoV-induced morbidity and mortality , and iii) MyD88-dependent signaling through a receptor(s) other than IL-1R1 or IL-18R1 is responsible for protection from rMA15.

### Increased viral loads and viral spread in the lungs of MyD88^−/−^ mice

To determine if lethal infection of MyD88-deficient mice was due to enhanced and/or prolonged virus replication, a kinetic analysis of rMA15 viral loads within the lungs of WT, RAG-1^−/−^, and MyD88^−/−^ mice was performed. Viral loads in lung tissue of rMA15-infected MyD88^−/−^ mice were significantly higher than WT mice at 2, 3, 4, and 6 dpi and RAG-1^−/−^ mice at both 2 and 4 dpi (Fig. 1C). Interestingly, despite the absence of significant signs of disease at late time points, viral lung titers remained elevated in lung tissue of rMA15-infected RAG-1^−/−^ mice at 7 and 9 dpi (Fig. 1C). In fact, no new or relapsing signs of disease were observed in rMA15-infected RAG-1^−/−^ mice as late as 5 weeks post-infection (data not shown). In addition to lung tissue, viral titers were also determined for the brain, liver, kidney, and spleen. Infectious virus was not detected in the brain, liver, or kidney of WT or MyD88^−/−^ mice at 1, 3, or 4 dpi (limit of detection = 250 pfu/gram of tissue; n = 4–5 mice per time point). Sporadic viral titers were detected in the spleens of WT and MyD88^−/−^ mice (data not shown). These findings indicate that the greater mortality observed in rMA15-infected MyD88^−/−^ mice was not due to enhanced replication at extrapulmonary sites.

**Figure 2:**
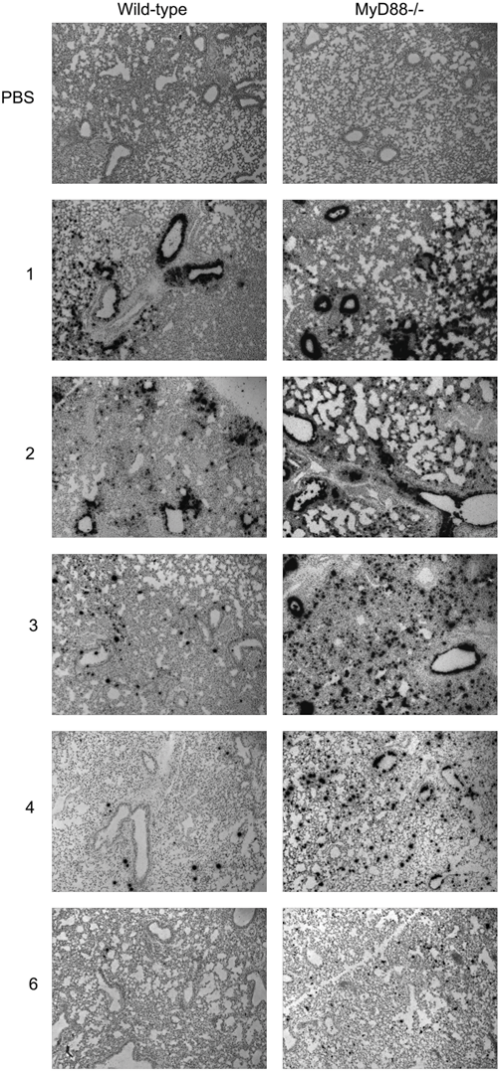
Virus replication is sustained and distribution is more widespread within the lungs of MyD88^−/−^ mice as compared to WT mice. 5 µM paraffin-embedded sections derived from the lung tissue of WT and MyD88^−/−^ mice were hybridized with an ^35^S-UTP-labeled riboprobe complementary to either the N gene of SARS-CoV (Urbani) or to the EBER2 gene from Epstein-Barr virus (data not shown). Images (magnification, 100×) are representative of at least three mice.

To further assess the role of MyD88 in controlling SARS-CoV replication within the lung, in situ hybridization was performed on tissue sections using an ^35^S-labeled riboprobe complementary for the N gene of SARS-CoV. As shown in Fig. 2, in situ signal was not observed in lung sections derived from mice that received intranasal administration of PBS alone (top panels). At both 1 and 2 dpi, intense rMA15-specific in situ signal was observed throughout the lung tissue, including lung airway epithelia, in rMA15-infected WT and MyD88^−/−^ mice. However, by 3 to 4 dpi, the distribution and intensity of rMA15-specific in situ signal had greatly diminished in lung tissue of WT mice, while the rMA15-specific signal in MyD88^−/−^ mice was more intense and much more broadly distributed (Fig. 2). By 6 dpi, though diminished, rMA15-specific in situ signal was still readily detectable in lung tissue of MyD88^−/−^ mice, whereas only very rare rMA15-specific in situ signal could be detected in lung tissue of WT mice. In sum, these findings suggest that MyD88 is required for control of MA15 replication in pulmonary tissue at early times post-infection and that the inability to control or clear this early replication is associated with increased lethality.

### Decreased expression of proinflammatory cytokines and chemokines in MyD88^−/−^ mice

**Figure 3:**
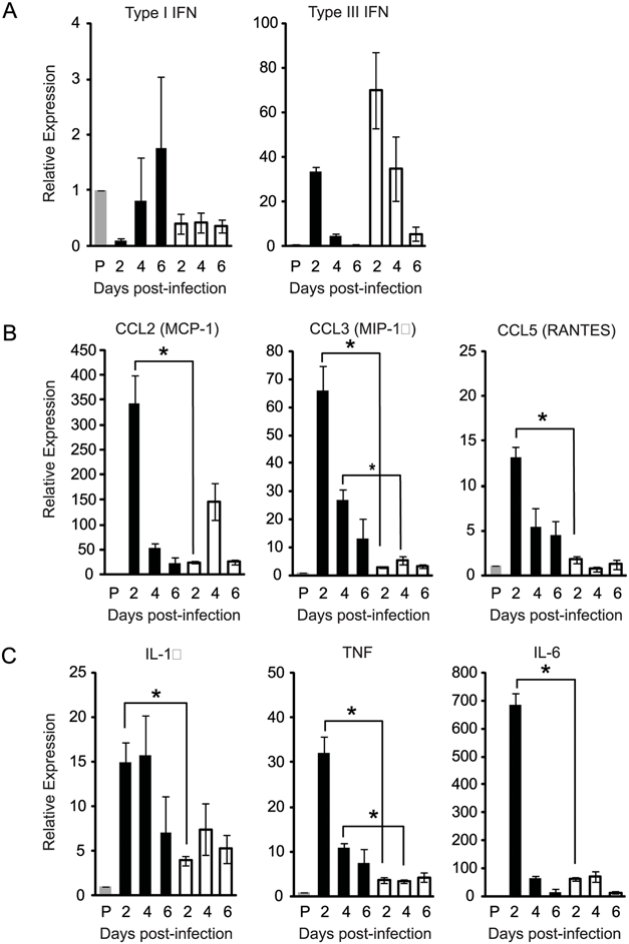
rMA15 induction of proinflammatory genes is reduced in MyD88^−/−^ mice. WT (black bars) and MyD88^−/−^ mice (white bars) were inoculated intranasally with PBS (grey bars) or 10^5^ pfu rMA15. At 2, 4, and 6 dpi, mice were euthanized and total lung RNA was analyzed for mRNA expression by qRT-PCR. Levels of gene transcription for Type I and Type III interferons (A), proinflammatory chemokines (B) and proinflammatory cytokines (C) were assessed. Data are normalized to 18S rRNA and are expressed as the relative fold increase over PBS inoculated mice. The data presented are the means from 3–4 mice per timepoint ± the standard error of the means. *, P<0.05.

Infection of WT mice with rMA15 results in a rapid inflammatory response in the lungs. This virus-induced inflammatory response likely has both protective and pathologic consequences. To investigate the importance of MyD88 in SARS-CoV-induced lung inflammation, we employed quantitative RT-PCR (qRT-PCR) to assess mRNA levels of antiviral and proinflammatory cytokine/chemokine gene expression in the infected mouse lung at various times post-infection. MyD88 is required for the induction of type I IFN in mouse cells following stimulation of TLR7 and TLR9 [14]. However, similar to previous reports, we were unable to detect significant induction of type I IFN in lung tissue of either WT or MyD88^−/−^ mice following SARS-CoV infection by qRT-PCR (Fig. 3A) or in serum using a type I IFN bioassay (data not shown) compared to mock-infected control mice [22]. Type III IFNs, which are induced by viral infection and TLR ligands, also have direct antiviral affects [23]. Recently, lung tissue and epithelial cells were found to be responsive to type III interferon in vivo, suggesting that type III IFN may function to prevent viral infection at mucosal surfaces [24],[25]. Interestingly, SARS-CoV infection of either WT or MyD88^−/−^ mice resulted in similar induction of type III IFN over mock-infected mice which peaked at 2 dpi and declined thereafter (Fig 3A). In fact, induction of type III IFN was slightly higher, although not statistically significant, in infected MyD88^−/−^ mice at all time points analyzed. Taken together, these findings suggest that the enhanced susceptibility of MyD88^−/−^ mice is not due to a failure to induce a protective type I IFN response, which was undetectable in both strains of mice, or type III IFN response, which was similar in both strains of mice.

Infection of WT mice with rMA15 resulted in a significant induction of proinflammatory chemokines including CCL2 (MCP-1), CCL3 (MIP-1α), and CCL5 (RANTES) as compared to mock-infected control mice (Fig. 3B). In contrast to type I and type III IFNs, induction of CCL2 was dramatically reduced in rMA15-infected MyD88^−/−^ mice compared to WT mice at 2 dpi (14 fold difference, P<0.005). Statistically significant differences in the abundance of CCL2 mRNA were not detected at 4 or 6 dpi (Fig. 3B). Similarly, the induction of CCL3 (22 fold difference at 2 dpi, P<0.005; 5 fold difference at 4 dpi, p<0.005) and CCL5 (8 fold difference at 2 dpi, P<0.005; 8 fold difference at 4 dpi, P<0.01) were dramatically reduced in infected MyD88-/- compared to infected WT mice (Fig. 3B). In addition to proinflammatory chemokines, virus-induced expression of several proinflammatory cytokines, including TNF-α (9 fold difference at 2 dpi, P<0.005; 3 fold difference at 4 dpi, p<0.005), IL-1β (3.8 fold difference at 2 dpi, P<0.01), and IL-6 (11 fold difference at 2 dpi, P<0.005); , was severely impaired in rMA15-infected MyD88^−/−^ mice compared to infected WT mice (Fig. 3C). These data indicate that MyD88 is required for the early induction of proinflammatory chemokines and cytokines within pulmonary tissues of SARS-CoV-infected mice and suggest that some aspect(s) of this inflammatory response is required for protection from lethal disease.

### Extensive lung pathology and delayed inflammation MyD88^−/−^ mice

**Figure 4:**
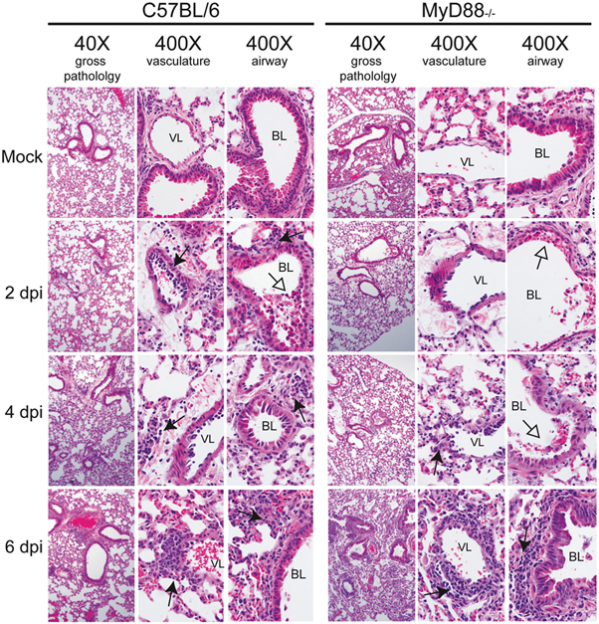
Lung pathology in rMA15 infected WT and MyD88^−/−^ mice. To assess lung damage and pulmonary inflammation throughout the course of virus infection, we evaluated hematoxylin and eosin stained lung tissue sections from 2, 4 and 6 dpi. WT mice at 2 dpi exhibited pronounced lung inflammation characterized by perivascular cuffing (filled arrowhead), endothelial and epithelial atypia (vacuolization and disruption of normal epithelium and endothelium), with minor denuding bronchiolitis (empty arrowhead) as seen in MyD88^−/−^ mice. Similar to what was seen at early times post infection, WT mice on 4 dpi show an exacerbation of the inflammatory infiltrate (filled arrowhead) seen on 2 dpi, PBV edema was not observed. By 6 dpi, normal bronchiolar epithelial architecture is restored but inflammatory infiltrates are still present. At 2 dpi, MyD88-/- mice exhibited a denuding bronchiolitis characterized by an extrusion of airway epithelial cells into the lumen of the airway (empty arrowhead) and epithelial/endothelial atypia, but did not exhibit any obvious signs of inflammation like peribronchivascular (PBV) or peri-venular immune cell infiltration. On 4 dpi, MyD88^−/−^ mice continue to exhibit a denuding bronchiolitis (empty arrowhead), epithelial/endothelial atypia, and now show PBV edema without a pronounced immune cell infiltration around the airway though perivascular cuffing was observed (filled arrowhead). Interestingly, by 6 dpi, the severity of PBV edema and denuding bronchiolitis in MyD88^−/−^ mice had waned, and signs of immune cell infiltration were evident, with marked PBV infiltrates and perivascular cuffing (filled arrowhead) even more severe than that seen in WT mice at similar times post infection. Overall, the host immune response to the virus was similar in quality and quantity, but delayed in onset by 5–6 days in the MyD88^−/−^ mice. Vascular and bronchiolar lumen are labeled as VL and BL, respectively.

The levels of inflammatory chemokine and cytokine transcription suggested that the innate immune response was severely delayed in MyD88-deficient mice as compared to WT mice. To assess lung damage and pulmonary inflammation throughout the course of virus infection in WT and MyD88^−/−^ mice, we evaluated hematoxylin and eosin stained lung tissue sections from 2, 4 and 6 dpi (Fig. 4). At 2 dpi, MyD88^−/−^ mice exhibited a denuding bronchiolitis characterized by an extrusion of airway epithelial cells into the lumen of the airway and epithelial/endothelial atypia (vacuolization and disruption of normal epithelium and endothelium) but did not exhibit any obvious signs of inflammatory cell infiltration including peribronchivascular (PBV) or peri-venular immune cell infiltration (“cuffing”). In contrast, WT mice at 2 dpi exhibited pronounced lung inflammation characterized by perivascular cuffing, endothelial and epithelial atypia, and peribronchivascular immune cell infiltration, without the severe denuding bronchiolitis seen in MyD88^−/−^ mice (Fig. 4). At 4 dpi, MyD88^−/−^ mice continued to exhibit a denuding bronchiolitis, epithelial/endothelial atypia, and the added phenotype of PBV edema without immune cell infiltration around the airway though perivascular infiltration of immune cells was observed (Fig. 4). Similar to what was seen at early times post infection, WT mice at 4 dpi had an exacerbation of the inflammatory infiltrate seen at 2 dpi, but denuding bronchiolitis and PBV edema were not observed. Interestingly, by 6 dpi, the severity of PBV edema and denuding bronchiolitis in MyD88^−/−^ mice had waned, and signs of lung inflammation were evident, with marked PBV infiltrates and perivascular cuffing even more severe than that seen in WT mice at similar times post infection. These findings suggest that MyD88 is essential for the early induction of the host inflammatory response and the timely recruitment of inflammatory leukocytes in the SARS-infected lung.

### Delayed recruitment of inflammatory monocytes to lungs of MyD88^−/−^ mice

The expression analyses of proinflammatory chemokines/cytokines and the lung pathology suggested that MyD88 is critical for early immune/inflammatory responses in lung tissue following SARS-CoV infection. To investigate whether the impaired chemokine and cytokine responses in MyD88^−/−^ mice impacted the cellular composition within the lung, total leukocytes were isolated from enzymatically digested pulmonary tissue and the cell surface phenotypes of the isolated cells were determined by flow cytometry.

**Figure 5:**
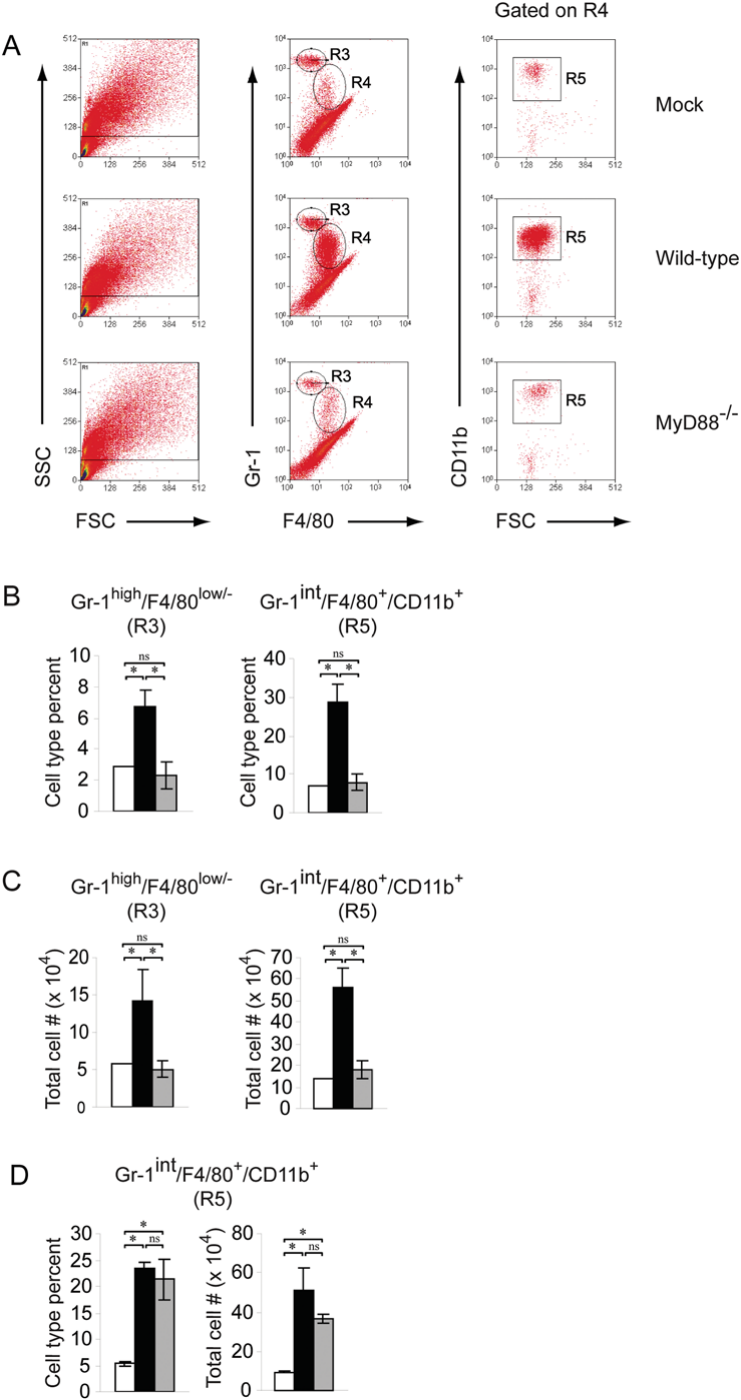
Recruitment of inflammatory monocytes/macrophages to the SARS-CoV infected lung is delayed in MyD88^−/−^ mice as compared to WT mice. 10 wk old B6 WT or MyD88^−/−^ mice were inoculated intranasally with PBS or 10^5^ pfu rMA15. (A) At 2 dpi, lung leukocytes were isolated as described in Materials and Methods and analyzed by flow cytometry. Histograms are representative of three mice. Two independent experiments gave similar results. (B) Percent inflammatory monocytes/macrophages of total lung leukocytes isolated from mock (□), rMA15-infected WT (▪), or rMA15-infected MyD88^−/−^ (▪) mice at 2 dpi. (C) Total numbers of inflammatory monocytes/macrophages isolated from mock (□), rMA15-infected WT (▪), or rMA15-infected MyD88^−/−^ (▪) mice at 2 dpi. (D) Percent inflammatory monocytes/macrophages of total lung leukocytes (left panel) and total numbers of inflammatory monocytes/macrophages (right panel) isolated from mock (□), rMA15-infected WT (▪), or rMA15-infected MyD88-/-n (▪) mice at 4 dpi.

At 2 dpi, no significant differences were detected in the number of natural killer cells (NK1.1^+^/CD3^−^) or T lymphocytes (CD4^+^/CD3^+^/NK1.1^−^ or CD8^+^/CD3^+^/NK1.1^−^) isolated from the lung tissue of mock-infected or SARS-CoV infected WT mice (data not shown). These findings suggested that the differences in inflammation in rMA15-infected WT and MyD88^−/−^ mice observed in the histological analyses of lung tissue were likely due to differences in myeloid cell populations. Therefore, anti-CD11b, anti-CD11c, anti-Gr-1, and anti-F4/80 antibodies were used to define the following cell types by cell surface antigen staining: alveolar macrophages (CD11c^+^/F4/80^+^/CD11b^low/−^), dendritic cells (CD11c^+^/CD11b^−^ or CD11b^+^/F4/80^−^/Gr-1^−^), inflammatory monocytes/macrophages (CD11b^+^/F4/80^+^/Gr-1^int^/CD11c^−^), and neutrophils (Gr-1^high^/CD11b^+^/F4/80^−^/CD11c^−^) [26]–[28]. As shown in Fig. 5A, cell surface staining of lung leukocytes with anti-Gr-1, anti-F4/80, and anti-CD11b antibodies revealed two distinct cell populations, defined by region 3 (R3) and R4/R5 in the histograms, that were significantly increased in both percentages (Fig. 5B) and total numbers (Fig. 5C) in rMA15-infected WT mice as compared to mock-infected mice. The cells defined by R3 in our analyses have a Gr-1^high^/F4/80^−^/CD11b^+^ cell surface phenotype (Fig. 5A and data not shown), which is consistent with that of neutrophils, and were modestly increased in the lung tissue of rMA15-infected WT mice at 2 dpi (Fig. 5B and 5C). The most dramatic differences detected in percentages and total numbers were cells with a Gr-1^int^/F4/80^+^/CD11b^+^ cell surface phenotype defined in R4 and R5 (Fig 5A, B, and C). Additional analyses demonstrated that these cells were Ly6C^+^ and CD11c^−^ (data not shown). This cell surface staining pattern, including the Gr-1^int^ and F4/80^low^ staining, has been well characterized by a number of studies as that of inflammatory monocytes [26]–[35]. Strikingly, both the Gr-1^high^/F4/80^−^ population (R3) and the Gr-1^int^/F4/80^+^/CD11b^+^ inflammatory monocyte/macrophage population (R4 and R5) were dramatically reduced in lung tissue of rMA15-infected MyD88^−/−^ mice compared to infected WT mice (Fig. 5A, B, and C). In fact, at 2 dpi, similar numbers of inflammatory monocytes were detected in infected MyD88^−/−^ and mock-infected control mice (Fig. 5C). To determine if the failure to recruit inflammatory monocytes/macrophages was sustained at later times post infection in MyD88^−/−^ mice, we performed similar cell isolation experiments at 4 dpi. In contrast to 2 dpi and consistent with our histological analysis of lung tissue, at 4 dpi, a similar percentage and total number of Gr-1^int^/F4/80^+^/CD11b^+^/CD11c^−^ inflammatory monocytes/macrophages were isolated from the lung tissue of WT and MyD88^−/−^ mice (Fig. 5D). Similar to 2 dpi, we did not detect significant numbers of CD3^+^ T lymphocytes within the lung tissue of rMA15-infected mice compared to PBS-inoculated control mice on 4 dpi, indicating that T lymphocytes were not a major component of the inflammatory response at these times post-infection (data not shown). These results further indicate that MyD88 is critical for early host immune and inflammatory responses, which include the initial recruitment of inflammatory monocytes/macrophages to pulmonary sites, in response to rMA15 infection.

### Chemokine receptors contribute to protection from mouse-adapted SARS-CoV infection

**Figure 6:**
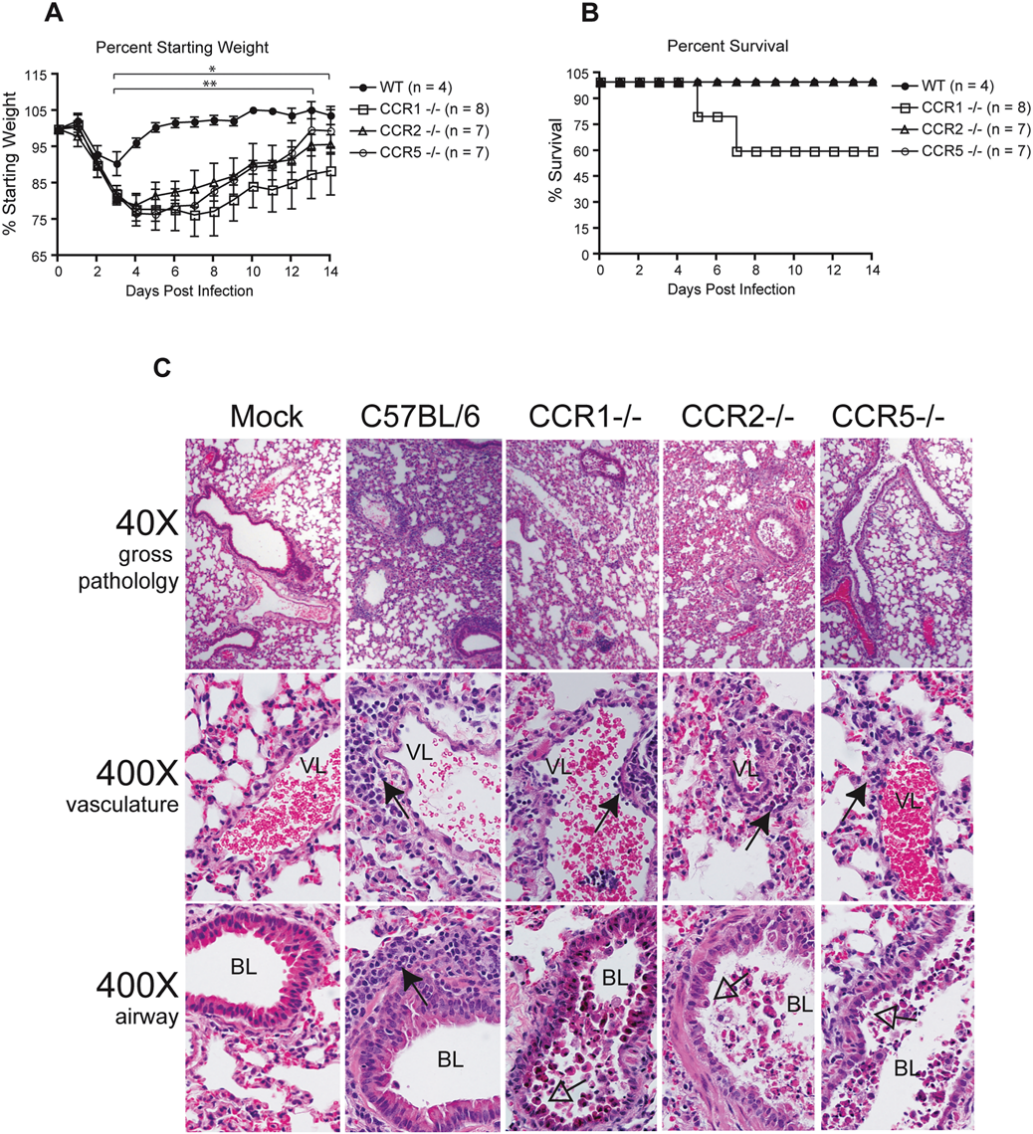
Chemokine receptors contribute to protection from mouse-adapted SARS-CoV infection. To assess the importance of chemokine receptors in SARS-CoV pathogenesis we infected CCR1, CCR2, and CCR5 deficient mice with a recombinant mouse adapted SARS-CoV (rMA15). (A) Mice were monitored for changes in body weight at 24 h intervals. Each data point represents the mean ± the standard deviation. On days 3–14 dpi (*), the differences in the weights of CCR1 and CCR2 mice as compared to WT mice were statistically different by Mann-Whitney test with a P value cutoff of 0.05. On days 3–13 dpi (**), the differences in the weights of CCR5 deficient and WT mice were statistically different by Mann-Whitney test with a P value cutoff of 0.05 (B) Mice were monitored for mortality and the data is expressed as percent survival. (C) To assess the lung damage and degree of pulmonary inflammation in chemokine receptor deficient mice, we evaluated hematoxylin and eosin stained lung sections from 2 dpi. Signs of inflammation and virus induced lung pathology are evident in wild-type mice on 2 dpi with peribronchivascular cuffing (filled arrowhead) caused by infiltrating immune cells, apoptosis of the airway epithelium, and a mild denuding bronchiolitis (empty arrowhead). In contrast, mice deficient in either CCR1, CCR2, or CCR5 exhibited more prominent airway epithelial cell apoptosis, a severe denuding bronchiolitis with an accumulation of cohesive apoptotic debris within the airway (empty arrowhead) , and perivenular/periarterial cuffing (filled arrowhead) but there was a distinct lack of cuffing around the affected airways. Bronchiolar and vascular lumen is labeled as “BL” and “VL”, respectively.

The histological and flow cytometric analyses outlined above suggested that monocytes/macrophages are i) the major cell population increased in the SARS-infected lung at early times post-infection, ii) increased in the lung by a MyD88-dependent mechanism, and iii) critical for protection against severe rMA15-induced disease. In addition, in response to rMA15 infection, MyD88^−/−^ mice failed to upregulate expression of a number of proinflammatory chemokines that promote monocyte recruitment. Therefore, we hypothesized that mice deficient in monocytes or specific chemokine receptors important for recruitment of inflammatory monocytes may be more susceptible to rMA15-induced disease. We attempted to deplete circulating monocytes by IP injection of clodronate liposomes or alveolar monocytes/macrophages by IN administration of clodronate liposomes in C57BL/6 mice prior to rMA15 infection. Though we were able to deplete alveolar macrophages by IN administration of clodronate liposomes, both IP and IN administration of clodronate liposomes failed to alter morbidity and mortality and failed to prevent the recruitment of inflammatory monocytes to the infected lung at 2 dpi (data not shown). Additionally, the intranasal administration of clodronate liposomes 2 days post rMA15 infection of C57BL/6 mice failed to induce more severe disease or mortality. Due to inconclusive results from our clodronate depletion studies and to continue to explore the importance of monocyte recruitment in SARS-CoV disease, we infected mice deficient in chemokine receptors known to be important for monocyte recruitment. As shown in Fig. 6A, mice deficient in CCR1, CCR2, or CCR5 developed more severe and prolonged disease as compared to WT mice. Between 3 and 14 dpi, CCR1 and CCR2 percent weight differed significantly from WT mice, while CCR5 weight differed significantly from WT between 2 and 13 dpi (Fig. 6A). Unlike CCR2 and CCR5 deficient mice, 40% of infected CCR1 deficient mice succumbed to infection by 7 dpi (Fig. 6B). To assess the lung damage and degree of pulmonary inflammation in chemokine receptor deficient mice, we evaluated hematoxylin and eosin stained lung sections from 2 dpi (Fig. 6C). Signs of inflammation and virus induced lung pathology are evident in wild-type mice on 2dpi with PBV cuffing caused by infiltrating immune cells, apoptosis of the airway epithelium, and a mild denuding bronchiolitis. In contrast, mice deficient in either CCR1, CCR2 or CCR5 exhibited more prominent airway epithelial cell apoptosis, a severe denuding bronchiolitis with an accumulation of cohesive apoptotic debris within the airway, and perivenular/periarterial cuffing but there was a distinct lack of cuffing around the affected airways. In direct correlation with the increased morbidity and mortality of rMA15 infected CCR1 deficient mice, the lung pathological conditions described above were the most severe in CCR1 deficient mice.

## Discussion

Since human clinical SARS data is complicated by host genetic variation, disease exacerbating comorbidities, age variation, and variable drug treatment regimens, animal models provide a more homogenous and controlled environment within which to ask questions related to the mechanisms of disease pathogenesis. Prior to the generation of a mouse adapted SARS-CoV (MA15) which causes 100% mortality in BALB/c mice, previous SARS-CoV BALB/c or C57BL/6 animal models using the epidemic strain, SARS Urbani, were purely models of in vivo virus replication without overt signs of disease [12],[13],[36],[37]. In contrast to previous models, our novel C57BL/6 model of SARS-CoV pathogenesis recapitulates disease similar to non-severe human SARS-CoV cases with high virus titer replication in the lung, significant weight loss, elevated inflammatory cytokine/chemokines, the recruitment of inflammatory cells to the lung, viral clearance and subsequent convalescence [4]–[6],[9]. Furthermore, the recovery from rMA15 disease is dependent on MyD88 but does not seem to be dependent on the presence of functional T or B cells (Fig. 1A). In contrast to a previous report where RAG-1^−/−^ mice were demonstrated to clear SARS Urbani (dose: 1×10^4^ TCID_50_) with similar kinetics as compared to WT mice, we demonstrate that RAG-1^−/−^ mice recover from disease signs with similar kinetics as WT mice but are unable to clear (dose: 10^5^ pfu) the more robust rMA15 [12]. The discrepancy regarding clearance of virus in RAG-1^−/−^ mice may be due to the differing doses used and/or the enhanced pathogenesis of the mouse adapted virus. The disease observed in our rMA15 C57BL/6 disease model has also been observed with a second independently derived mouse adapted SARS-CoV suggesting that the disease phenotype is not simply an artifact of the rMA15 mutational spectra but that both sets of mouse adapting mutations enhance the intrinsic pathogenic potential of the epidemic strain (data not shown). Taken together, the morbidity and mortality data for rMA15 infected WT, RAG-1^−/−^, and MyD88^−/−^ mice suggest that early MyD88 dependent innate signals are required for protection from rMA15 induced mortality.

Serological and pathological data from the SARS-CoV epidemic suggests that the innate immune response plays a crucial role in the control of SARS-CoV infection but the molecular mechanisms of innate immune activation, protection from severe disease, and the contribution of the innate response to immune pathology remain unknown [5],[9],[10],[38]. MyD88 is a key signaling adaptor protein for most all TLRs, IL-1R1, IL-18R1, and IFNγ-R1 [14]. Contrary to previous virological studies [16]–[19],[21],[39], we have demonstrated MyD88 plays a crucial role in protection from SARS-CoV infection independent of Type I (α/β) and III (IL-28/29 or interferon lambda) interferon, and the adaptive immune response. Though MyD88 mediated proinflammatory signaling has been implicated in the protection from numerous bacteria and parasitic infections, few in vivo studies have implicated MyD88 in protection from viral diseases [16]–[19], [21], [40]–[44]. Intranasal infection of MyD88-deficient mice with RSV or VSV produces more severe disease that was correlated with a failure to recruit immune cells to the sites of infection [17],[18]. For RSV, MyD88-dependent induction of type I interferon correlated with the recruitment of eosinophils to the lung and efficient virus clearance [17]. In contrast, MyD88-dependent protection from lethal VSV infection occurred independent of type I interferon, correlated with the recruitment of monocytes to the site of infection and was dependent on IL-1R1 signaling [18]. In the C57BL/6 mouse model of SARS-CoV pathogenesis reported here, we demonstrate MyD88-mediated protection from SARS-CoV infection in the absence of detectable induction of type I interferon. Furthermore, infection of IFNα/β receptor deficient mice with rMA15 results in moderate weight loss and complete recovery with kinetics that is indistinguishable from those of WT mice (personal communication, Frieman and Baric, manuscript in preparation). Unlike RSV and VSV, we found that WT C57BL/6 mice are protected from lethal SARS-CoV infection by a MyD88-dependent mechanism that does not involve adaptive immunity, the induction of type I/III interferon, or IL1-R/IL-18R signaling (data not shown) suggesting that SARS-CoV is interfacing with the innate immune system in a potentially novel manner.

Human cases of SARS-CoV, mouse models, and in vitro data suggest inflammatory chemokines and cytokines and the recruitment of inflammatory cells are important in SARS-CoV pathogenesis [5],[10]. Our studies indicate that protection from SARS-CoV infection correlates with MyD88-dependent induction of IL1-β, TNF-α, IL-6, MCP-1, MIP-1α, and RANTES at early times post infection and many of these cytokines/chemokines were upregulated in human SARS-CoV cases [9],[45]. At early times post rMA15 infection, the MyD88-dependent chemokine/cytokine response occurs with the recruitment of inflammatory monocytes/macrophages to the lung at 2 dpi and is coincident with the control of virus replication in WT animals. Days 2, 3, 4 and 6 post infection, virus titers are significantly lower in WT mice as compared to MyD88^−/−^ animals and these data are supported by the dramatic loss of in situ hybridization signal in WT mice by 3 dpi. Furthermore, the lung pathology and flow cytometry results suggest that the absence of inflammation in MyD88^−/−^ mice at early times post infection (2 dpi) correlates with much more severe lung damage and by the time the host mounts an adequate inflammatory response (4 dpi), lung damage is too severe and mortality ensues. The importance of macrophages in SARS-CoV pathogenesis has been noted in the past where SARS antigen was frequently detected in macrophages in the pathological evaluation of post mortem lung tissues from human SARS-CoV cases [11]. Interestingly, in vitro data suggests that macrophages are not productively infected by SARS-CoV, however, these cells secrete inflammatory cytokines like IP-10 and MCP-1 in response to the virus [46]. We have yet to determine the cell type responsible for the induction of the MyD88-dependent protective inflammatory response though we have demonstrated the recruitment of inflammatory monocytes/macrophages occurs even if alveolar macrophages are depleted in WT mice (data not shown). In the future, bone marrow chimeras between WT and MyD88^−/−^ mice may help deduce if myeloid derived cells are responsible for the initial induction of the protective inflammatory response.

Chemokine receptors play a crucial role in directing inflammatory leukocytes to the sites of infection in order to mount an effective immune response [28],[47]. CCR1, CCR2 and CCR5 each bind a unique repertoire of chemokine ligands but all are able to guide the trafficking of monocytes and other leukocytes to sites of inflammation [28],[47]. Previous reports have implicated that that chemokine receptors, CCR1, CCR2 and CCR5 can both promote protection (CCR1,CCR2) and progression (CCR5) of disease caused by a neurovirulent coronavirus and the chemokine receptor dependent alteration of disease correlated with the recruitment of inflammatory leukocytes to the sites of infection [48]–[50]. Our studies demonstrate the importance of chemokine receptors in protection from rMA15 disease where CCR1, CCR2 and CCR5 deficient mice experienced a significantly more severe disease and associated mortality as compared to WT mice. Furthermore, infected CCR deficient mice suffered from severe lung pathology (denuding bronchiolitis, epithelial apoptosis, etc.) and defects in inflammatory cell recruitment to the airway that were very similar to those seen in MyD88^−/−^ mice. CCR1 (MCP-1), CCR2 (MIP-1α) and CCR5 (MIP-1α and RANTES) bind chemokines upregulated in the lungs of rMA15 infected WT mice whose expression are coincident with the recruitment of inflammatory leukocytes to the lung and protection from mortality. Recent data from Glass et al. suggest that CCR5 dependent recruitment of monocytes, T cells and NK cells to the brains of West Nile virus infected WT mice are required for the control of virus replication in the CNS and protection from mortality [51]. CCRs can also guide immunopathogenesis during virus infection where CCR2 deficient mice are protected from a lethal influenza virus infection due to the failure to recruit inflammatory monocytes to the infected lung [27]. Taken together, the above data suggests an important role for MyD88-dependent inflammation, the innate immune response, and chemokine recruitment of inflammatory cells in both the prevention and progression of severe SARS-CoV disease.

Viral pathogenesis is a complex process where interactions between the virus and the host determine the outcome of virus-induced disease. Many of the pathogenic mechanisms of SARS-CoV disease remain unknown and the existence of a robust mouse model of SARS-CoV pathogenesis will allow for the detailed analysis of virus-host interactions. We have developed a novel model of acute SARS-CoV pathogenesis. Using this model, we discovered a critical role for MyD88-dependent inflammation in the protection from SARS-CoV induced mortality suggesting that the innate immune response plays a key role in the early control of SARS-CoV in the lung. Our future studies are aimed at understanding the mode of MyD88 dependent innate immune activation and the molecular mechanisms of inflammatory monocyte clearance of SARS-CoV from the lung. In the future, our studies may guide epidemiological studies in human populations in order to deduce if MyD88 related inflammatory genes or CCRs contributed to protection or prevention of severe SARS-CoV disease. Importantly, our SARS-CoV disease model can be employed to study the contributions of various innate immune genes in the protection from severe SARS-CoV disease which eventually may help clarify the current view of SARS-CoV pathogenesis and guide the development of intelligently designed antiviral therapies.

## Materials and Methods

### Viruses and Cells

Vero E6 cells were grown in MEM (Invitrogen, Carlsbad, CA) supplemented with 10% FCII (Hyclone, South Logan, UT) and gentamycin/kanamycin (UNC Tissue Culture Facility). Stocks of the recombinant mouse-adapted SARS-CoV (rMA15) were propagated and titered on Vero E6 cells and cryopreserved at −80°C until use as described [52]. All viral and animal experiments were performed in a Class II biological safety cabinet in a certified biosafety level 3 laboratory containing redundant exhaust fans while wearing personnel protective equipment including Tyvek suits, hoods, and HEPA-filtered powered air-purifying respirators (PAPRs) as described [52].

### Mice

C57BL/6J (stock# 000664) , RAG-1^−/−^ (stock# 002216), IL-1R1^−/−^ (stock# 003245), and IL-18R^−/−^ (stock# 004131) mice were obtained from The Jackson Laboratory (Bar Harbor, Maine) and bred in house. MyD88^−/−^ mice were obtained from Shizou Akira (Osaka University) and backcrossed 11 generations to the C57BL/6 background. CCR1^−/−^, CCR2^−/−^, CCR5^−/−^, and control C57BL/6 were obtained from Taconic Laboratories. Animal housing and care were in accordance with all UNC-Chapel Hill Institutional Animal Care and Use Committee guidelines. 10 week old mice were anaesthetized with a mixture of ketamine/xylazine and intranasally infected with either DPBS alone or 10^5^pfu/50 µl rMA15 or the recombinant epidemic strain, icSARS, in DPBS (Invitrogen, Carlsbad, CA). Mice were monitored at 24 h intervals for virus-induced morbidity and mortality.

### Viral tissue and serum titers

To quantify the amount of infectious virus in tissues, lung, liver, kidney, spleen, and brain tissue were weighed, placed in 0.5 ml DPBS, homogenized, and titered via plaque assay on Vero E6 cells as previously described [53]. Whole blood was harvested via cardiac puncture and collected in BD microtainer tubes for serum separation. Serum was titered via plaque assay as described above.

### Histological Analysis

Lung tissues were fixed in PBS/4% paraformaldehyde, pH 7.3, tissues were embedded in paraffin, and 5 µm sections were prepared by the UNC histopathology core facility. To determine the extent of inflammation, sections were stained with hematoxylin and eosin (H & E) and scored in a blinded manner.

### In situ hybridization

^35^S-UTP-labeled riboprobes specific to the N gene of SARS-CoV (Urbani) or to the EBER2 gene from Epstein-Barr virus (negative control probe) were generated with an SP6-specific MAXIscript in vitro transcription kit (Ambion) and in situ hybridization was performed as described previously [37]. Briefly, deparaffinized tissue sections were hybridized with 5×10^4^ cpm/µl of ^35^S-labeled riboprobes overnight. Tissues were washed, dehydrated through graded ethanol, coated in NTB autoradiography emulsion (Kodak), and incubated at −80°C for 7 days. Following development, sections were counterstained with hematoxylin and silver grain deposition was analyzed by light microscopy. rMA15-specific signal was determined by comparing silver grain deposition on parallel sections hybridized with the ^35^S-labeled riboprobe complementary for the EBER2 gene of Epstein-Barr virus.

### qRT-PCR

Lungs from mock- or rMA15-infected mice were removed and homogenized directly in 1 ml of Trizol reagent (Invitrogen) and total RNA was isolated following the manufacturer's instructions. Complementary DNA was generated from 0.25–1 ug of total RNA using 250 ng random primers (Invitrogen) and superscript III reverse transcriptase (Invitrogen). Real-time PCR experiments were performed using Taqman© gene expression assays and an AB Prism 7300 (Applied Biosystems). 18S rRNA was used as an endogenous control to normalize for input amounts of cDNA. The relative fold induction of amplified mRNA were determined by using the Ct method.

### Flow cytometry

Mice were inoculated as described above, sacrificed by exsanguination at 2 and 4 days post-infection, and lungs were perfused via cardiac puncture with 1× PBS. Lungs were dissected, minced, and incubated for 2 hours with vigorous shaking at 37°C in digestion buffer [RPMI, 10% FBS, 15 mM HEPES, 2.5 mg/ml collagenase A (Roche), 1.7 mg/ml DNase I (Sigma)]. Cells were passed through a 40 micron cell strainer, resuspended in RPMI media, layered on 5 ml lympholyte-M (Cedarlane), and centrifuged 30 minutes at 2500 rpm. Banded cells were collected, washed in wash buffer (1× HBSS, 15 mM HEPES), and total viable cells were determined by trypan blue exclusion. Isolated cells were incubated with anti-mouse FcγRII/III (2.4G2; BD Pharmingen) for 20 min. on ice and then stained in FACS staining buffer (1× HBSS, 1% FBS, 2% normal rabbit serum) with the following antibodies from eBioscience: anti-F4/80-FITC, anti-Gr-1-PE, anti-CD11b-APC, anti-CD11c-PE, anti-Ly-6C-FITC, anti-CD3-FITC, anti-CD8-APC, anti-CD4-PerCP, and anti-NK1.1-PE. Cells were fixed overnight in 2% paraformaldehyde and analyzed on a Cyan cytometer (Dako) using Summit software.

### Statistical analyses

Percent starting weights, viral titers and inflammatory cell numbers were evaluated for statistically significant differences by the non-parametric Mann-Whitney test within GraphPad Prism or unpaired t-tests using GraphPad InStat3 software. P values of ≤0.05 were considered significant.
